# Effects of Composite Resin Teeth Versus Porcelain Teeth in Complete Dentures on Oral Health-Related Quality of Life, Masticatory Function, and Patient Satisfaction: A Randomized Controlled Trial

**DOI:** 10.3390/dj14020088

**Published:** 2026-02-03

**Authors:** Asuka Kodama, Toshifumi Nogawa, Yoshiyuki Takayama, Kiwamu Sakaguchi, Atsuro Yokoyama

**Affiliations:** Department of Oral Functional Prosthodontics, Division of Oral Functional Science, Graduate School of Dental Medicine, Hokkaido University, Sapporo 060-0813, Japan; a.kodama@den.hokudai.ac.jp (A.K.); takayama@den.hokudai.ac.jp (Y.T.); sakaguti@den.hokudai.ac.jp (K.S.); yokoyama@den.hokudai.ac.jp (A.Y.)

**Keywords:** artificial teeth, composite resin, denture, complete, mastication, porcelain, dental

## Abstract

**Background/Objectives**: Artificial teeth in complete dentures are classified according to the materials used: porcelain (PO) or composite resin (CR). However, these materials’ effects on function, patient satisfaction, and quality of life (QOL), as well as occlusal wear, remain unclear. We compared PO and CR complete dentures in edentulous patients by assessing masticatory function, patient satisfaction, and oral health-related QOL at 3, 6, and 12 months post-insertion, as well as occlusal surface morphology owing to material differences. **Methods**: In this open-label, randomized, single-center, parallel-group study, participants were edentulous patients who visited our hospital and underwent treatment with new complete dentures. The outcomes were oral health-related QOL; subjective satisfaction, assessed using a visual analog scale; and masticatory performance, evaluated with gummy jelly and were assessed at baseline and 3, 6, and 12 months post-denture insertion. Occlusal surface impressions were taken twice, digitized as STL models, superimposed, and analyzed for wear. The Wilcoxon rank-sum test was used to compare between groups. **Results**: All evaluated items showed improvement. However, no significant differences were observed between the PO and CR groups, including between the amount of wear observed in the two groups. However, the PO group showed a tendency toward less wear. Extended observation may be required to clarify the long-term effects of artificial tooth materials. **Conclusions**: In the short term, the artificial tooth material did not influence masticatory function, oral health-related QOL, or patient satisfaction.

## 1. Introduction

Declining masticatory function affects social life, daily activities, and mental well-being in older adults, and edentulous individuals are more likely to experience malnutrition [1]. However, undernutrition improves with use of complete dentures [2]. Daily functioning can also improve with the use of removable dentures [3]. Therefore, dentures are important to maintaining quality of life (QOL) among older adults [4].

Complete dentures comprise a denture base and artificial teeth, with base materials being made of denture resin or metal. Commonly used materials for artificial teeth in complete dentures include acrylic resin (AR), composite resin (CR), and porcelain (PO). AR teeth chemically bond to the denture base but are susceptible to wear. CR teeth containing fillers have superior wear resistance and esthetic advantages owing to their multilayer structure, handling ease, and appropriate hardness for occlusal adjustment [4]. A comparison of hardness between AR and CR teeth revealed that the hardness increases with greater filler contents and larger filler sizes [4].

PO teeth are used clinically for their high wear resistance, sustained esthetics over time, and low plaque accumulation [5,6]. However, they are susceptible to impact, occlusal noise related to a high degree of hardness [7], and poor adhesion to the denture base resin. Clinical use of PO teeth has declined in recent years, now representing only 2% of all artificial teeth that are shipped [7]. Because of the inherent difficulty in adjusting PO teeth, only a limited number of products have been commercially available, resulting in restricted options for comparison. However, the recent introduction by Shofu of a product with an identical form has made direct comparison possible. Owing to its wear resistance, the occlusal height is less likely to change over time. Its stain resistance may also help maintain its esthetic appeal in the long term, which could benefit patients.

The extent of wear and morphological changes in clinical settings, as well as differences between PO and composite resin teeth, remain unclear. Demonstrating that ceramic teeth exhibit less morphological change than hard resin teeth over time would confirm the stability of PO teeth.

Studies have investigated the mechanical properties of artificial teeth, such as their hardness and color stability [5,6], as well as oral health-related QOL among denture wearers, including the effects of impression techniques [8,9] and occlusal schemes [10,11]. However, no studies have examined the effects of artificial tooth materials on satisfaction and oral health-related QOL among complete denture wearers. The extent of wear and clinical morphology changes during clinical use, as well as differences between composite resin and PO teeth, remain unclear. Factors associated with wear include sex, age, and age-related muscle weakness [12]. Differences in artificial tooth hardness can affect the wear resistance and durability of dentures, which affects the long-term prognosis [5].

This study aimed to evaluate the subjective and objective effects of different artificial tooth materials when used in complete dentures on masticatory function, patient satisfaction, and oral health-related quality of life in edentulous patients, from a patient-centered perspective. We also investigated the extent of occlusal surface morphology changes owing to differences in artificial tooth materials when used in complete dentures. To investigate the stability of PO as a material used for complete dentures, we examined the degree of occlusal surface morphological changes resulting from differences in artificial tooth materials. The null hypothesis was that no difference would exist in masticatory function, patient satisfaction, or oral health-related QOL between the CR and PO groups at 3, 6, and 12 months after insertion of complete dentures.

## 2. Materials and Methods

### 2.1. Study Design

We conducted an open-label, randomized, parallel-group trial at a single center. Participants were randomized using block randomization, with a block size of six, based on a random number table. Identification numbers were assigned sequentially according to the order of enrollment, and participants were allocated to each group within each block using a pre-specified allocation table. We included patients who visited our hospital between February 2018 and March 2025 and underwent complete denture treatment. Inclusion criteria were age ≥20 years, use of complete dentures in both jaws, clinical assessment by a dentist indicating the need for new complete dentures for both jaws, and provision of written informed consent. Exclusion criteria were categorization as Class IV under the classification system for prosthodontic treatment of edentulous patients of the Japan Prosthodontic Society [13], presence of jaw defects, severe xerostomia, insufficient interalveolar ridge distance to accommodate PO teeth (<10 mm), inability to continue regular hospital visits, or determination by the investigators that participation was otherwise inappropriate.

This study was conducted in accordance with the Declaration of Helsinki and approved by the Ethical Review Board for Life Science and Medical Research at Hokkaido University Hospital (No. 017-0359) and the Hokkaido University Certified Review Board (Approval 018-016, jRCT number: jRCTs012180009; date of approval 13 February 2018). This randomized clinical trial was conducted and reported in accordance with the CONSORT guidelines. The completed CONSORT checklist is provided as supplementary Table S1.

**Figure 1:**
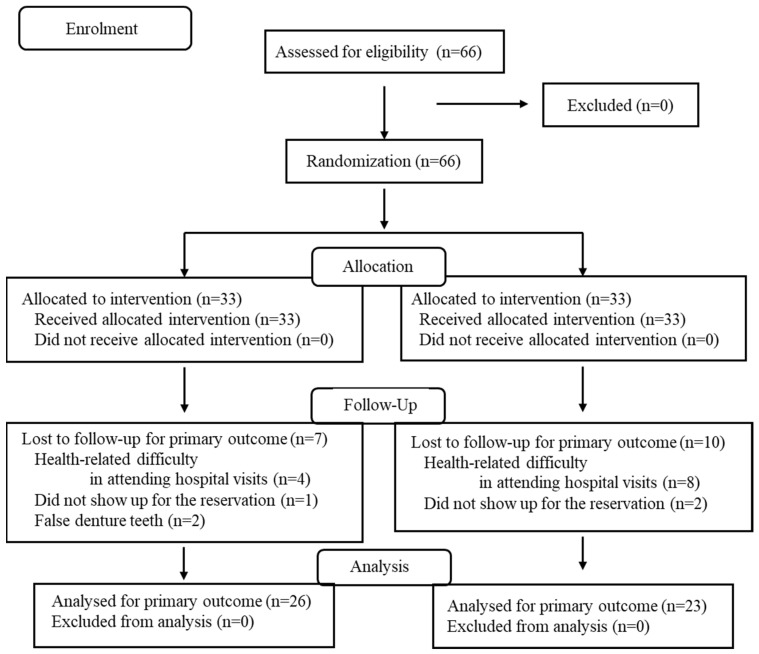
Study inclusion flowchart.

Eligible patients meeting the inclusion criteria were enrolled and randomly assigned to receive either PO or CR dentures. Dentures were fabricated using a standard method by the attending dentist [14]. For participants whose dentures were fabricated using the conventional method, the procedure included preliminary and definitive impressions, jaw relationship records using occlusal rims, trial insertion, and final delivery of the complete dentures. Following treatment, post-insertion reviews and necessary adjustments were performed. This study was conducted as an open-label trial. The type of artificial teeth was disclosed to attending dentists as they were producing jaw relationship records and to participants at the time of denture insertion. As the treating dentist, patient, and evaluator could identify which artificial teeth had been assigned to each person by looking at the denture, it was difficult to perform a blind test. Border molding was conducted using a custom tray with a compound; impressions were then taken with polyvinyl silicone material. Maxillomandibular registration was obtained using occlusal rims and silicone bite registration material. CR (Veracia SA, Shofu Inc., Kyoto, Japan [15]) or PO (Veracia SA Porcelain, Shofu Inc., Kyoto, Japan) teeth—identical in shape—were selected for new dentures. Dental technicians performed tooth arrangement. The appropriate occlusal scheme (fully bilateral balanced occlusion or lingualized occlusion) was determined by the attending dentist. After confirming the occlusal relationship, esthetics, and phonetics with trial dentures, the final dentures were completed and delivered. Evaluation was conducted on both previous dentures and new dentures, after 3, 6, and 12 months of use, by a dentist other than the individual in charge of treatment (Figure 1).

### 2.2. Participant Information

We collected information on patients’ age, sex, systemic medical history, oral history, residual ridge morphology (evaluated using treatment difficulty indices for edentulous patients developed by the Japan Prosthodontic Society [16,17]), condition of denture wear, panoramic radiograph findings, and denture hygiene status. Evaluation items were oral health-related QOL, degree of satisfaction, and masticatory performance.

#### 2.2.1. Oral Health-Related QOL

The Japanese version of the Oral Health Impact Profile (OHIP-J) was used [18]. The OHIP-J, which includes the 19-item OHIP-EDENT-J module for patients without teeth, has been shown to be reliable and valid, making it suitable for assessing quality of life related to oral health. Based on responses to the OHIP-J, we extracted items corresponding to the Japanese version of the OHIP for edentulous patients (OHIP-EDENT-J) and recorded scores [19]. It has been suggested that a difference of approximately 10 points in the OHIP-EDENT score represents a clinically meaningful change in oral health-related quality of life.

#### 2.2.2. Degree of Satisfaction

A visual analog scale (VAS) was used to assess overall satisfaction, esthetics, mastication, pronunciation, pain, discomfort, and stability.

#### 2.2.3. Masticatory Performance

Participants were instructed to chew a gummy jelly (Glucolum^®^, GC, Tokyo, Japan) for 20 s and then rinse with 10 mL of water and spit all contents—gummy jelly, water, and saliva—into a filter cup. The amount of glucose that was released during mastication was measured. Each parameter was assessed before fabrication of the new dentures (baseline), and then again at 3, 6, and 12 months after denture insertion [20,21].

#### 2.2.4. Clinical Wear of Denture Teeth

The amount of wear on the artificial teeth was evaluated. Participants were defined as those who completed the 12-month evaluation after denture placement. Impressions were taken at any point more than 1 year after denture placement (1) and again 1 year later (2); the amount of wear on the occlusal surfaces of artificial teeth was compared.

Impressions of the occlusal surfaces of patients’ dentures were taken using silicone material (Silde fit, Shofu Inc., Kyoto, Japan) to make plaster models. To assess morphology, the occlusal surface of the artificial teeth was scanned using the Artec micro industrial scanner (Data Design Co., Ltd., Aichi, Japan) to generate STL data. The two types of STL scan data (1 and 2) were overlaid using Geomagic Wrap software. After overlaying all dentition STL data, each tooth was segmented individually and re-overlaid. Buccal and lingual surfaces of the artificial teeth, which are less affected by time-related wear, were prioritized to approach a distance of 0 mm. The Free Form Modeling System was used to compare the wear volumes of 1 and 2.

### 2.3. Statistical Analysis

The required sample size was calculated using a two-sided significance level (α) of 5%, power of 80%, and common standard deviation of 11.6, assuming that a 10-point difference in the OHIP-EDENT score indicated that the PO teeth were superior [22]. The sample size calculation was based on an expected standardized effect size of Cohen’s d = 0.83, with equal variances being assumed between the groups. This calculation resulted in 23 participants per group, with a target of 46 participants. The dropout rate in 2021 was 19%; the primary factor in dropouts was suspension of follow-up visits during the COVID-19 pandemic. Estimating the dropout rate at 20%, the target was increased to 26 participants per group (total of 52); the final target number of participants for primary evaluation was set to 66.

Background information on participants was analyzed using *t*-tests. The primary endpoint of this study was the OHIP-EDENT-J score at 3M. Comparisons among baseline and 3-, 6-, and 12-month scores for both the PO and CR groups were performed using the Wilcoxon signed-rank test. Effect sizes were calculated as r = Z/√N and interpreted according to Cohen’s criteria. A *t*-test was used to analyze artificial tooth wear.

JMP 18 (SAS Institute Inc., Cary, NC, USA) was used for statistical analysis. The significance threshold was set at 0.05.

Participants with missing data were excluded.

This study was funded by a contract research study commissioned by the author from SHOFU INC.

## 3. Results

Initially, 66 individuals consented to study participation, but 17 dropped out before the 3-month evaluation. A total of 49 participants (17 men, 32 women) completed the 3-month assessments: 26 in the CR group and 23 in the PO group. Between the 3- and 6-month assessments, 9 participants dropped out, meaning that 40 participants (14 men, 26 women) completed 6-month assessments: 19 in the CR group and 21 in the PO group.

A total of 12 participants dropped out between the 6- and 12-month assessments, leaving 28 participants (10 men, 18 women) who completed the 12-month assessments: 13 in the CR group and 15 in the PO group. The study flowchart is presented in Figure 1.

Background information regarding study participants is shown in Table 1.

The results for OHIP, masticatory performance, and VAS at 3, 6, and 12 months are presented in Table 2 and Table 3. No significant differences were observed in any of the evaluated items.

**Figure 2:**
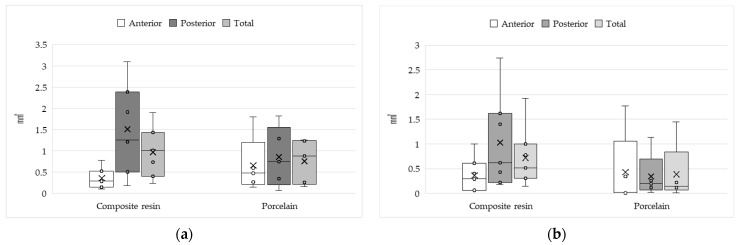
Amount of wear to artificial teeth: (**a**) maxillary complete denture; (**b**) mandibular complete denture. Figure legend: ×,mean; ◦, individual data points.

Figure 2 shows the amounts of wear, with no significant differences being observed.

## 4. Discussion

Although informed consent was obtained from more participants than the calculated target sample size to account for potential dropouts, the declaration of a state of emergency owing to Coronavirus disease 2019 (COVID-19) led some patients to avoid hospital visits. Consequently, the dropout rate exceeded our expectations.

We used randomized allocation and adjusted for confounding factors. The artificial teeth used—Veracia SA for CR and Veracia SA Porcelain for PO—were identical in shape, enabling evaluation of material differences without the influence of morphological variation. The artificial tooth material did not considerably alter oral health-related QOL, masticatory performance, or VAS scores at 3, 6, and 12 months after denture insertion (Table 2 and Table 3).

A strong relationship exists between OHIP scores and patient satisfaction [23,24], and psychological factors exert a substantial influence on OHIP outcomes [25]. OHIP-EDENT was designed to assess negative denture-related impacts [26]. We observed improvements across all scores in both the PO and CR groups, indicating the positive effect of new dentures [27]. However, no significant differences in OHIP-EDENT-J or VAS scores were found between the groups at any measurement points. Kakuta et al. suggested that patients’ oral health-related QOL is best explained by the interaction of multiple factors, although they did not mention artificial tooth materials [23]. Additional analyses of other items are warranted in future.

The average reported OHIP-EDENT scores are 19 ± 15.99 at 8 weeks after denture insertion [26]. This aligns with the OHIP-EDENT-J scores observed at 3 months in our study. Dhadet et al. reported significant improvement in oral health-related QOL at 6 months post-denture fitting [25]. Although the observation periods differed, OHIP-EDENT scores generally improved during the initial stages of our study.

No significant differences in esthetics were observed at any measurement period. Patient-perceived esthetics is a key factor influencing satisfaction with complete dentures [5,28]. Although the degree of color change varies depending on the artificial tooth material (e.g., AR or nano-fCR) [5], such changes generally fall within clinically acceptable limits [29]. Ayaz et al. reported that discoloration in acrylic or reinforced acrylic teeth results from both absorption and adsorption of colorants; in PO teeth, only adsorption occurs [6]. We found no esthetic differences between the CR and PO groups. Although differences in water absorption and translucency exist between PO and CR, such that PO exhibits greater translucency, the short duration of our study presumably limited the detection of noticeable changes. When subjectively evaluating esthetics, factors beyond color change (including shape and alignment) may influence patient satisfaction.

We found no significant difference in the amount of wear between PO and CR. Generally, differences between PO and CR teeth include hardness, water absorption, wear resistance, esthetics (e.g., translucency), and technical difficulty in occlusal adjustment. PO reportedly has the smoothest surface and highest hardness among AR, CR, PO, and nanohybrid composites [5], with hardness and surface roughness being presumed to be related to artificial tooth wear. The degree of this wear is expected to vary based on denture-wearing duration, potentially altering the occlusal contact area over time [30]. PO teeth are considered more wear-resistant than CR ones, which may result in a longer denture adaptation period. Garg et al. reported that hardness is a critical property influencing wear resistance and denture durability. Specifically, AR’s lower hardness is associated with increased wear, whereas PO’s higher hardness contributes to greater wear resistance [5]. However, we found no significant difference between wear amounts of PO and CR, even after 12 months. This result would be influenced by the short 12-month duration and by the fact that the artificial CR teeth used herein are classified as micro-filled hybrid composite teeth, with a harder material than conventional CR [31].

Masticatory performance is related to the occlusal contact area [32,33]. Mello et al. evaluated clinical abrasion resistance in various denture teeth, including PO, and found no significant differences in wear among materials [34]. We did not measure the actual wear or occlusal contact area of the artificial teeth, but no significant difference in masticatory performance was observed between groups. Therefore, it was difficult to determine whether these factors influenced the masticatory performance of the dentures. However, the non-significant difference in wear between PO and CR is likely related to there being no difference in masticatory performance, which led to no differences in QOL. Future research should include long-term studies to determine whether differences in artificial tooth materials impact the occlusal contact area. Objective evaluations, such as bite force measurements, should also be considered.

Given that PO is made from a harder material, occlusal adjustment is technically more difficult. However, after 3 months of denture use, proper occlusal adjustment was achieved; sufficient stability was likely established.

Our study findings indicated that wearing new complete dentures improves oral health-related QOL. The short-term effect of artificial tooth material on improving oral health-related QOL was equivalent, as no clinically significant difference was observed between the PO and CR groups within 3 months after denture insertion. With respect to esthetics at fitting, both the PO and CR groups demonstrated improvement. Although PO teeth are generally considered superior in terms of esthetics, no clear difference was evident within 3 months after insertion.

### Limitations

As mentioned at the beginning of the discussion, the number of dropouts was higher than expected due to COVID-19. As noted by Forgie et al., patients who are treated at university hospitals may experience greater difficulty with denture adaptation than those who are treated in private dental clinics [22]. Our participants’ characteristics were consistent with that report; patients tended to express higher levels of dissatisfaction and stronger preferences regarding their dentures. Given the material properties of PO teeth, a longer-term evaluation is warranted. The sample size and follow-up duration were limited, and we relied on patient-reported outcomes, which may affect the generalizability of the results.

## 5. Conclusions

There was no significant difference in oral health-related QOL between the PO and CR groups after 3 months of wearing complete dentures. The null hypothesis was not rejected.

More long-term research is required.

## Figures and Tables

**Table 1:** Characteristics of study participants.

		Porcelain Teeth			Composite Resin teeth		
		3M	6M	12M	3M	6M	12M
		N = 23	N = 21	N = 15	N = 26	N = 19	N = 13
**Sex**	Male	9	8	5	8	6	5
	Female	14	13	10	18	13	8
**Age**	Mean (SD)	76.26 (9.48)	77.33 (9.72)	79.2 (10.56)	72.92 (8.93)	72.05 (9.22)	72.38 (7.98)
**Classification system for prosthodontic treatment**	1	16	14	10	19	13	9
	2	6	6	4	6	5	3
	3	1	1	1	1	1	1

Abbreviations: SD, standard deviation; 3M, 3 months after denture insertion; 6M, 6 months after denture insertion; 12M, 12 months after denture insertion

**Table 2:** OHIP and masticatory performance results at 3, 6, and 12 months after denture insertion.

			3M			6M			12M		
			Mean	SD	95%CI	Mean	SD	95%CI	Mean	SD	95%CI
**OHIP**	**EDENT-J score**	Porcelain teeth	20.17	13.87	14.18–26.17	18.62	13.07	12.67–24.57	16.40	10.34	10.67–22.13
		Composite resin teeth	18.12	12.24	13.17–23.05	16.37	8.81	12.12–20.61	16.85	8.99	11.4–22.27
		*p*	0.59			0.73			0.93		
		r	0.07			−0.05			0.02		
	**OHIP-49 summary score**	Porcelain teeth	44.83	32.24	30.88–58.77	41.14	31.25	26.25–55.37	37.80	25.61	23.6–251.98
		Composite resin teeth	40.35	27.51	29.23–51.36	36.47	20.49	26.60–46.34	40.69	21.18	27.89–53.50
		*p*	0.70			0.96			0.78		
		r	0.05			−0.009			0.05		
	**Psychosocial impact**	Porcelain teeth	12.35	12.1	7.12–17.58	10.48	11.18	5.39–15.56	10.93	8.88	6.02–15.85
		Composite resin teeth	8.81	9.51	4.97–12.65	8.47	8.40	4.43–12.52	10.54	7.74	5.86–15.22
		*p*	0.47			0.82			0.91		
		r	0.10			−0.04			−0.02		
	**Oral function**	Porcelain teeth	12.61	7.73	9.27–15.95	11.29	8.3	7.51–15.06	9.67	5.88	6.41–12.92
		Composite resin teeth	12.65	7.14	9.77–15.54	11.58	5.60	8.88–14.28	12.46	6.06	8.80–16.13
		*p*	1.0			0.54			0.26		
		r	0			0.10			0.21		
	**Oro-facial Appearance**	Porcelain teeth	4.83	4.05	3.07–6.58	4.81	4.72	2.66–6.96	4.87	5.03	2.08–7.65
		Composite resin teeth	3.85	3.44	2.46–5.23	3.42	2.8	2.07–4.77	4.08	2.93	2.31–5.85
		*p*	0.40			0.52			0.93		
		r	0.12			−0.10			−0.02		
	**Oro-facial pain**	Porcelain teeth	6.91	5.31	4.61–9.21	11.29	8.3	4.08–8.49	5.4	3.5	3.46–7.34
		Composite resin teeth	6.54	4.99	4.53–8.55	11.58	5.6	3.83–6.91	5.38	4.21	2.84–7.93
		*p*	0.98			0.72			0.93		
		r	−0.002			−0.06			0.02		
**Masticatory performance**		Porcelain teeth	91.44	43.62	72.57–110.30	90.74	33.62	75.43–106.04	88.33	52.72	59.14–117.53
		Composite resin teeth	94.90	40.52	78.54–111.27	98.24	44.91	76.59–119.88	87.81	33.05	67.83–107.78
		*p*	0.95			1.0			0.50		
		r	−0.009			0			0.13		

Abbreviations: 3M, 3 months after denture insertion; 6M, 6 months after denture insertion; 12M, 12 months after denture insertion; OHIP-49 summary score, Oral Health Impact Profile for edentulous patients; OHIP, Oral Health Impact Profile; EDENT-J score, Japanese version of the Oral Health Impact Profile for edentulous patients; SD, standard deviation.

**Table 3:** VAS results at 3, 6, and 12 months after denture insertion.

			3M			6M			12M		
			Mean	SD	95%CI	Mean	SD	95%CI	Mean	SD	95%CI
**VAS**	**Comprehensive**	Porcelain teeth	78.49	22.48	68.77–88.21	76.14	25.40	64.58–87.71	78.45	19.18	67.83–89.07
		Composite resin teeth	81.69	20.76	73.30–90.07	81.89	18.66	72.90–90.88	88.82	9.31	83.20–94.44
		*p*	0.57			0.69			0.13		
		r	−0.08			0.06			0.29		
	**Aesthetic**	Porcelain teeth	80.74	19.65	72.24–89.24	81.75	19.76	72.76–90.74	79.26	20.34	67.99–90.52
		Composite resin teeth	81.96	22.37	72.92–91.0	87.73	14.81	80.18–94.46	83.00	15.23	73.81–92.21
		*p*	0.50			0.43			0.84		
		r	−0.10			0.01			0.04		
	**Mastication**	Porcelain teeth	77.43	19.79	68.88–85.99	73.65	29.06	60.42–86.87	76.09	19.02	65.56–86.63
		Composite resin teeth	72.49	27.41	61.41–83.56	78.32	23.41	67.04–89.61	79.05	19.96	67.0–91.11
		*p*	1.00			0.79			0.71		
		r	0			0.04			0.07		
	**Pronunciation**	Porcelain teeth	75.65	20.93	66.60–84.70	76.85	26.95	64.58–89.12	76.77	19.04	66.22–87.31
		Composite resin teeth	76.16	21.39	67.52–84.80	80.33	17.33	71.98–88.68	80.37	14.88	71.38–89.36
		*p*	0.77			0.89			0.73		
		r	−0.04			−0.02			0.007		
	**Pain**	Porcelain teeth	75.76	23.98	65.39–86.13	75.94	25.06	64.53–87.34	77.44	21.59	65.49–89.39
		Composite resin teeth	69.63	26.68	58.85–80.40	81.99	18.53	73.06–90.92	87.89	9.01	82.50–93.38
		*p*	0.43			0.71			0.27		
		r	0.11			0.06			0.21		
	**Discomfort**	Porcelain teeth	73.16	25.21	62.26–84.06	73.48	24.84	62.17–84.79	78.99	19.39	68.25–89.73
		Composite resin teeth	72.65	22.73	63.47–81.83	81.94	14.99	74.72–89.17	83.24	14.61	74.41–92.07
		*p*	0.78			0.37			0.82		
		r	0.04			0.14			0.04		
	**Stability**	Porcelain teeth	77.04	21.94	67.56–86.53	76.06	23.62	65.31–86.81	76.7	18.82	66.28–87.12
		Composite resin teeth	75.08	23.82	65.46–84.70	81.52	18.92	72.40–90.65	83.17	16.2	73.38–92.96
		*p*	0.80			0.65			0.39		
		r	0.04			0.07			0.16		

Abbreviations: 3M, 3 months after denture insertion; 6M, 6 months after denture insertion; 12M, 12 months after denture insertion; VAS, visual analog scale.

## Data Availability

The data presented in this study are available on reasonable request from the corresponding author.

## Supplementary Materials

The following supporting information can be downloaded at: https://www.mdpi.com/article/10.3390/dj14020088/s1, Table S1: The completed CONSORT checklist. Ref. [35] is cited in Supplementary Materials.

## Abbreviations

The following abbreviations are used in this manuscript:

QOL	quality of life
AR	acrylic resin
CR	composite resin
PO	porcelain
OHIP-J	Japanese version of the Oral Health Impact Profile
OHIP-EDENT-J	OHIP for edentulous patients
VAS	visual analog scale
3M	3 months after wearing dentures
6M	6 months after wearing dentures
12M	12 months after wearing dentures
Coronavirus disease 2019	COVID-19
